# Skin characteristics of photoaging and intrinsic aging in Southwest Chinese population: a reflectance confocal microscopy and histopathological study

**DOI:** 10.3389/fphys.2026.1848754

**Published:** 2026-06-10

**Authors:** Guoxiu Cao, Yanjie Chen, Shengdong Guan, Lin Chen, Rentao Yu, Aijun Chen

**Affiliations:** 1 Department of Dermatology, The First Affiliated Hospital of Chongqing Medical University, Yuzhong, Chongqing, China; 2 Department of Dermatology, Guizhou Aerospace Hospital, Zunyi, Guizhou, China

**Keywords:** Asian skin, dermal collagen, dermo-epidermal junction, intrinsic skin aging, photoaging, reflectance confocal microscopy

## Abstract

Despite widespread use of reflectance confocal microscopy (RCM) in dermatology, normative aging data for Chinese populations remain limited. This study aimed to analyze the characteristics of skin microstructure under RCM and histomorphological differences between photoaging and intrinsic aging in Southwest Chinese adults, and to explore potential effect modification by occupational ultraviolet exposure. A total of 150 healthy volunteers (50 per group: 18–35, 45–55, and ≥65 years) underwent RCM imaging of the right zygoma and left volar forearm. Fifteen parameters were measured, such as epidermal thickness (ET), furrow patterns, irregular honeycomb, mottled pigmentation, collagen score (higher = greater degeneration), and papillary ring score. SCINEXA scores and work environments were recorded. Perilesional skin tissues from the face and forearm of young and elderly subjects were collected for histopathological analysis of epidermal thickness, rete ridge length, and collagen-positive area. ET showed site-specific variation characteristics: the volar forearm was significantly thinner in the older versus younger group (P<0.001), whereas facial skin showed an apparent nonlinear distribution, with greater thickness in the middle-aged versus younger group (49.0 ± 8.35 vs. 45.3 ± 5.44 μm; P = 0.021) and attenuation in the older group (42.0 ± 6.44 μm; P = 0.045). Collagen scores differed by site and age group: forearm scores exceeded facial scores in the younger group (forearm: 2.000 [1.000–4.000] vs. face: 1.00 [0.000–2.000]; P = 0.0003), whereas facial scores surpassed forearm scores in the middle-aged and older groups (face: 8.000 [7.000–8.250] vs. forearm: 7.000 [6.000–8.000]; P = 0.002). Facial polycyclic rings increased across age groups, from 4% in younger to 50% in older subjects (P<0.001). Outdoor work was associated with more pronounced facial aging markers in middle-aged and older versus younger subjects (collagen loss: P = 0.011–0.003; papillary flattening: P = 0.020). Histological findings were consistent with RCM observations. In this cross-sectional cohort study, site-specific differences in age-group distributions of skin aging-related parameters were observed. These RCM-derived structural parameters may facilitate differentiation between intrinsic aging and photoaging features; however, their potential value for informing aging intervention strategies requires validation through prospective interventional studies.

## Introduction

Skin aging represents a multifactorial process involving intrinsic (chronological) degeneration and extrinsic (environmental) damage, with ultraviolet radiation (UVR) accounting for approximately 80% of facial aging factors (Lu et al., 2022). While histopathology remains the diagnostic gold standard for assessing epidermal atrophy, dermo-epidermal junction (DEJ) flattening, and elastic fiber degeneration, its invasive nature and sampling bias limit longitudinal monitoring and cosmetic applications.

Reflectance confocal microscopy (RCM) enables non-invasive, quasi-histological imaging of skin at cellular resolution (0.5–1 μm lateral resolution) to depths of 250–350 μm, capturing the full epidermal thickness and superficial dermis (Shahriari et al., 2021). Current FDA-approved indications include skin tumor margin assessment and inflammatory dermatoses (Ardigo et al., 2016); however, emerging applications in quantitative aging assessment and treatment monitoring remain underutilized in Asian populations.

Ethnic variations in skin aging phenotypes are well-documented. Fitzpatrick phototype III–IV skin (predominant in East Asian populations) exhibits delayed wrinkle formation but prolonged post-inflammatory hyperpigmentation compared with type I–II skin (Kutlu Haytoglu et al., 2014). Previous RCM studies comparing Brazilian and French cohorts demonstrated earlier onset of photoaging in Brazilian women, suggesting population-specific thresholds for anti-aging interventions (Mercurio et al., 2016). However, existing RCM normative databases predominantly derive from Caucasian subjects, limiting generalizability to the Chinese. According to Roland Berger calculations, China’s aesthetic medicine market will reach nearly ¥370 billion in 2025, with market volume calculated to reach approximately ¥700 billion by 2030 (Roland Berger and Meituan Medical Beauty, 2025).

Critical knowledge gaps persist regarding: (i) the chronology of structural degradation in Chinese skin; (ii) differences in the rate of aging between facial photoaging and intrinsic aging in non-exposed areas; and (iii) the quantitative impact of occupational versus recreational UV exposure. This study establishes baseline RCM parameters across three adult age decades, evaluates the correlation between confocal parameters and clinical SCINEXA scores, determines whether UV exposure thresholds vary by age, and confirms the structural findings through histological validation.

## Materials and methods

### Study design and population

This single-center, cross-sectional observational study enrolled 150 healthy Chinese volunteers (Fitzpatrick phototypes II–IV) between October and December 2025 at Chongqing Medical University. Subjects were stratified into three age cohorts: young (18–35 years), middle-aged (45–55 years), and older (≥65 years), with well-matched sex distribution (P = 0.353).

Exclusion criteria included previous aesthetic procedures, pregnancy, chronic immunosuppression, or systemic diseases affecting skin integrity. Specific procedural exclusions required the following washout periods: topical retinoid use within 3 months; laser, intense pulsed light, or chemical peel within 6 months; oral isotretinoin within 12 months; and systemic hormonal therapy (including hormone replacement therapy) within 3 months. The protocol followed Declaration of Helsinki guidelines and was approved by the Institutional Ethics Committee of the First Affiliated Hospital of Chongqing Medical University.

### RCM image acquisition

Imaging utilized the Vivascope 1500 (Caliber Imaging & Diagnostics, Rochester, NY; 830 nm laser, 30 mW power, 0.5×0.5 to 8×8 mm field of view). Following cleansing and alcohol defatting, immersion gel was applied. The depth reference (Z = 0) was calibrated at the first visible honeycomb pattern (stratum spinosum). Z-stacks were acquired at 2 μm increments to the papillary dermis (depth ~200–300 μm). The capture sites included the right zygomatic region (chronically sun-exposed) and the mid-volar left forearm (occasionally exposed). At each site, a 2×2 mm composite image was captured at four levels: stratum corneum, mid-spinous layer, DEJ, and papillary dermis.

Z-stacks were acquired by trained operators who were blinded to participants’ exact age during image capture. RCM image analysis was performed offline by observers who were blinded to both the anatomic site of acquisition and participants’ ages. Two observers jointly reviewed and reached consensus through joint discussion for all images. Technical adequacy was assessed immediately after acquisition. Images: no significant motion artifact covering >10% of the image area. Any Z-stack failing to meet these criteria was reacquired at the same session until a technically adequate image was obtained. No technically inadequate images were included in the final analysis.

### Clinical and confocal parameters

SCINEXA Assessment: Validated 23-item scale (5 intrinsic + 18 extrinsic features) graded 0–3 by two independent dermatologists (inter-rater κ=0.84).

Work Environment: Binary classification based on occupational UV exposure >50% of working hours (08:00–16:00).

RCM Quantification:

1. Epidermal Thickness (ET): Distance from the first honeycomb (excluding the stratum corneum) to the first collagen signal (DEJ), measured in three fields and averaged.
2. Furrow Patterns: Categorized as: (a) small rhomboidal (minor diagonal ≤1mm), (b) large rhomboidal (>1mm), (c) disarranged, (d) linear, or (e) absent.
3. Irregular Honeycomb & Mottled Pigmentation: Semiquantitative scale (0=absent, 1=<10%, 2 = 10–50%, 3=>50% of field area).
4. Collagen Score (0–12): Calculated as Σ(coverage grade × type coefficient), where coverage=0–4 (25% increments) and type coefficients were thin reticulated (0), coarse (1), huddled (2), curled (3).
5. Papillary Ring Score (0–12): Same algorithm using morphological categories: regular (0), irregular (1), absent (2), polycyclic (3).

### Histological examination: H&E and Masson’s trichrome

Normal perilesional skin tissues were collected from the faces and forearms of young and elderly subjects. Specimens were obtained from patients undergoing cosmetic excision of benign melanocytic nevi or seborrheic keratoses at Chongqing Medical University. Biopsies were performed incidental to the primary cosmetic procedure with additional informed consent. Specimens were harvested from clinically normal-appearing skin >0.5 cm from the lesion margin, confirmed by preoperative dermatoscopic assessment. The lesions were histologically confirmed as benign melanocytic nevi post-excision. Lesions with clinical or dermatoscopic suspicion of malignancy, or specimens showing inflammatory or neoplastic pathology on histopathologic review were excluded.

The specimens were fixed in 10% neutral buffered formalin for 24 hours, dehydrated through a graded ethanol series, embedded in paraffin, and sectioned at 6 μm. Hematoxylin and eosin (H&E) staining was performed to examine epidermal morphological structure, while Masson’s trichrome staining was employed to evaluate collagen fiber morphology and distribution. Quantitative analysis was conducted using ImageJ software, measuring epidermal thickness and epidermal rete ridge height. Six samples were obtained for each group, with five random fields per sample selected for analysis. Additionally, the percentage of collagen-positive areas in Masson’s trichrome staining was quantified.

### Statistical analysis

Statistical analyses were performed using GraphPad Prism version 10.0. Descriptive statistics included absolute/relative frequencies for categorical variables. Descriptive statistics included absolute/relative frequencies for categorical variables. Normally distributed continuous variables were presented as mean ± standard deviation (SD), while non−normally distributed continuous and ordinal semiquantitative RCM scores were reported as median (interquartile range, IQR). Normally distributed continuous variables were analyzed using one-way ANOVA with Tukey’s honestly significant difference (HSD) for multiple pairwise comparisons or independent t-tests for two-group comparisons. Paired comparisons between face and forearm were conducted using paired t-tests. Non-normally distributed continuous variables and ordinal composite scores were analyzed using the Kruskal-Wallis H test with Dunn’s Bonferroni adjustment for multiple pairwise comparisons or the Mann-Whitney U test for two-group comparisons. Paired comparisons were conducted using Wilcoxon signed-rank tests. Categorical and correlation analyses. Categorical variables were analyzed using chi-square or Fisher’s exact tests. Correlations were assessed using Pearson or Spearman correlation coefficients. A two-tailed P-value < 0.05 was considered statistically significant for overall comparisons.

## Results

### Baseline characteristics

Groups were well-matched for sex, phototype distribution, and occupational exposure (**Table 1**). SCINEXA scores demonstrated expected age stratification (intrinsic: 1.0 [0.0, 2.0] vs. 7.0 [6.0, 8.0] vs. 9.0 [8.0, 10.0]; extrinsic: 1.0 [0.0, 2.0] vs. 10.0 [9.0, 12.0] vs. 19.0 [16.8, 22.0]; both P < 0.001). All of them had excellent compliance with the RCM examination, and no subject was excluded.

**Table 1:** Baseline clinical characteristics of study participants.

Characteristic	Younger(n=50)	Middle-age (n=50)	Older (n=50)
Age (median IQR)	25.5 (20.0, 30.0)	49.5 (47.0, 52.0)	70.0 (67.0, 74.0)
Sex			
man	19.0 (38.0%)	16.0 (32.0%)	23 (46%)
woman	31.0 (62.0%)	34.0 (68.0%)	27 (54%)
Fitzpatrick Phototype			
II	2.0 (4.0%)	3.0 (6.0%)	1.0 (2.0%)
III	34.0 (68.0%)	31.0 (62.0%)	37.0 (74.0%)
IV	14.0 (28.0%)	16.0 (32.0%)	12.0 (24.0%)
Work Environment			
Indoor	33.0 (66.0%)	30.0 (60.0%)	22.0 (44.0%)
Outdoor	17.0 (34.0%)	20.0 (40.0%)	28.0 (56.0%)
SCINEXA (medianIQR)			
Intrinsic SCINEXA	1.0 (0.0, 2.0)	7.0 (6.0, 8.0)	9.0 (8.0, 10.0)
Extrinsic SCINEXA	1.0 (0.0, 2.0)	10.0 (9.0, 12.0)	19.0 (16.8, 22.0)
Total SCINEXA	2.0 (1.0, 4.0)	16.0 (15.0, 21.0)	28.5 (25.0, 31.0)

### Epidermal architecture

Thickness Across Age Strata: On the volar forearm, median epidermal thickness was 42.0 (38.0–47.0) μm in the young group, 40.5 (36.0–45.3) μm in the middle group (P = 0.237 vs young, r=0.144) and 36.0 (33.0–41.0) μm in the older group (P<0.001 vs young, r=0.425; P = 0.003 vs middle-aged, r=0.271). On the face, thickness values differed across age strata: the middle-aged group had thicker epidermis than young participants (49.0 ± 8.35 vs. 45.3 ± 5.44 μm; P = 0.021, d=0.54), while older group had thinner epidermis than young participants (42.0 ± 6.44 μm; P = 0.045, d=0.48). Middle−aged participants also had thicker epidermis than older group (P < 0.0001, d = 1.02).

**Figure 1:**
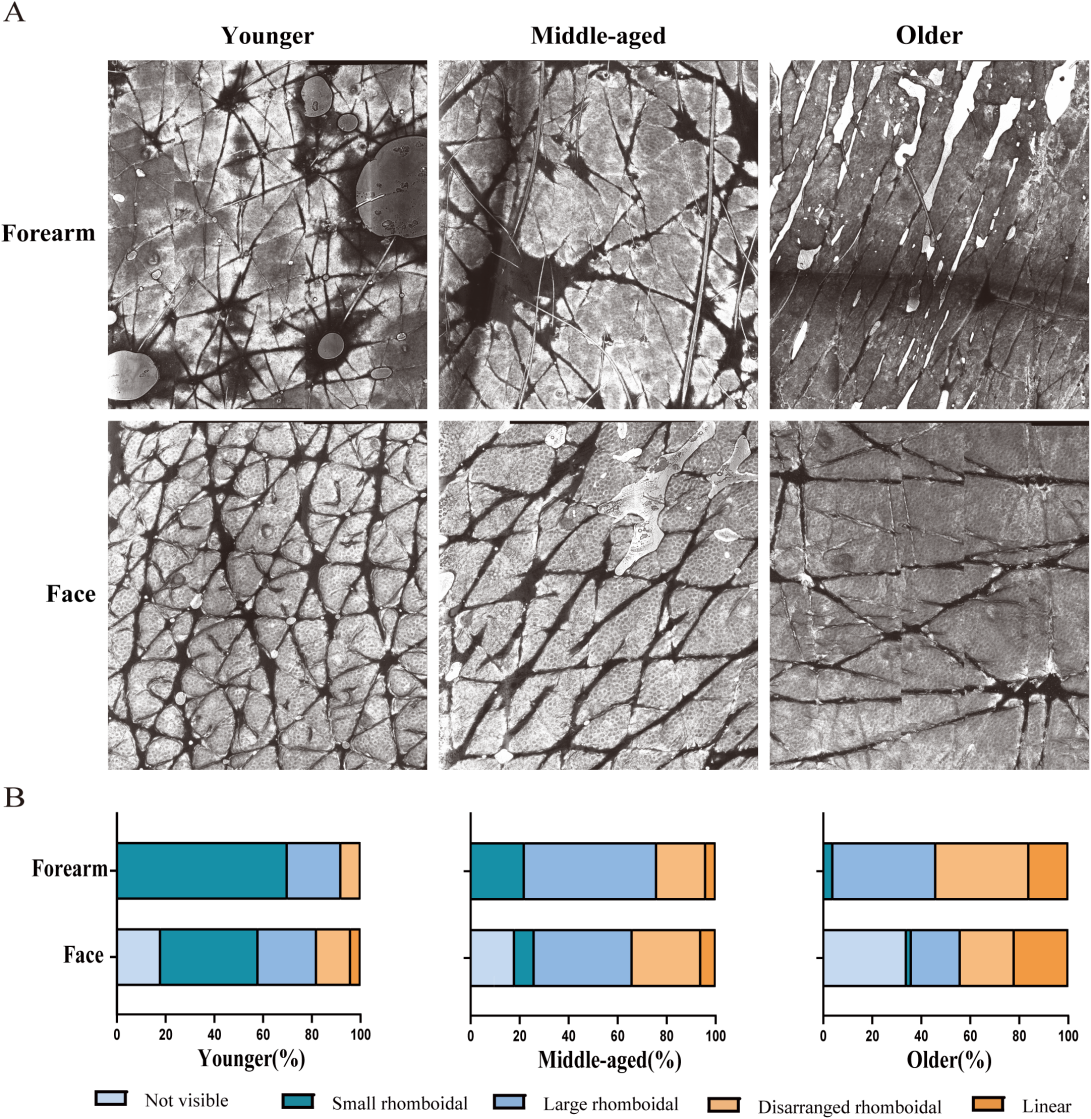
Skin furrow patterns. **(A)** Representative RCM images of furrow patterns across age groups and anatomic sites. Younger subjects show small rhomboidal patterns on both sites; middle-aged subjects show large rhomboidal patterns; older subjects demonstrate linear patterns on the face and disarranged patterns on the forearm. **(B)** Three percentage-stacked bar charts corresponding to young, middle-aged, and older age strata (n = 50 per group). x-axis = percentage (0–100%).); The y-axis displays anatomic sites (face, forearm). Small rhomboidal (Rhom.), Large rhomboidal (Large rhom.), Disarranged rhomboidal (Disarr. rhom.), Linear furrow pattern (Linear), Without visible furrow (Not visible).

Surface Morphology: Furrow pattern distribution differed by age group and anatomic site. Small rhomboidal patterns were more prevalent in the young group (forearm 70%, face 40%), while large or disarranged patterns were more common in the middle-aged group (forearm 54% large rhomboidal structure, face 28% disarranged). Linear structure and furrow absence are more common in the older group (face: 34% absent); notably, absent furrows were not observed on the forearm at any age. On the face, absent furrows were present across all age groups (young, 18%; middle, 18%; and older, 34%). Examples of furrow patterns are shown in **Figure 1**.

### Pigmentary and cellular changes

Mottled Pigmentation: Peak prevalence and severity were observed in middle−aged facial skin (94% prevalence vs. 30% in young participants, with 60% of middle−aged subjects showing >10% field involvement). Older skin exhibited lower severity (78% prevalence, 28% with >10% involvement). Median scores were 0.00 (0.00–1.00) in the young, 2.00 (1.00–3.00) in the middle-aged, and 1.00 (1.00–2.00) in the older participants. Middle-aged severity was significantly higher than young (P < 0.0001, r = 0.54); older severity was significantly higher than young (P = 0.0037, r = 0.26), and middle-aged severity was significantly higher than older (P = 0.0026, r = 0.27).

**Figure 2:**
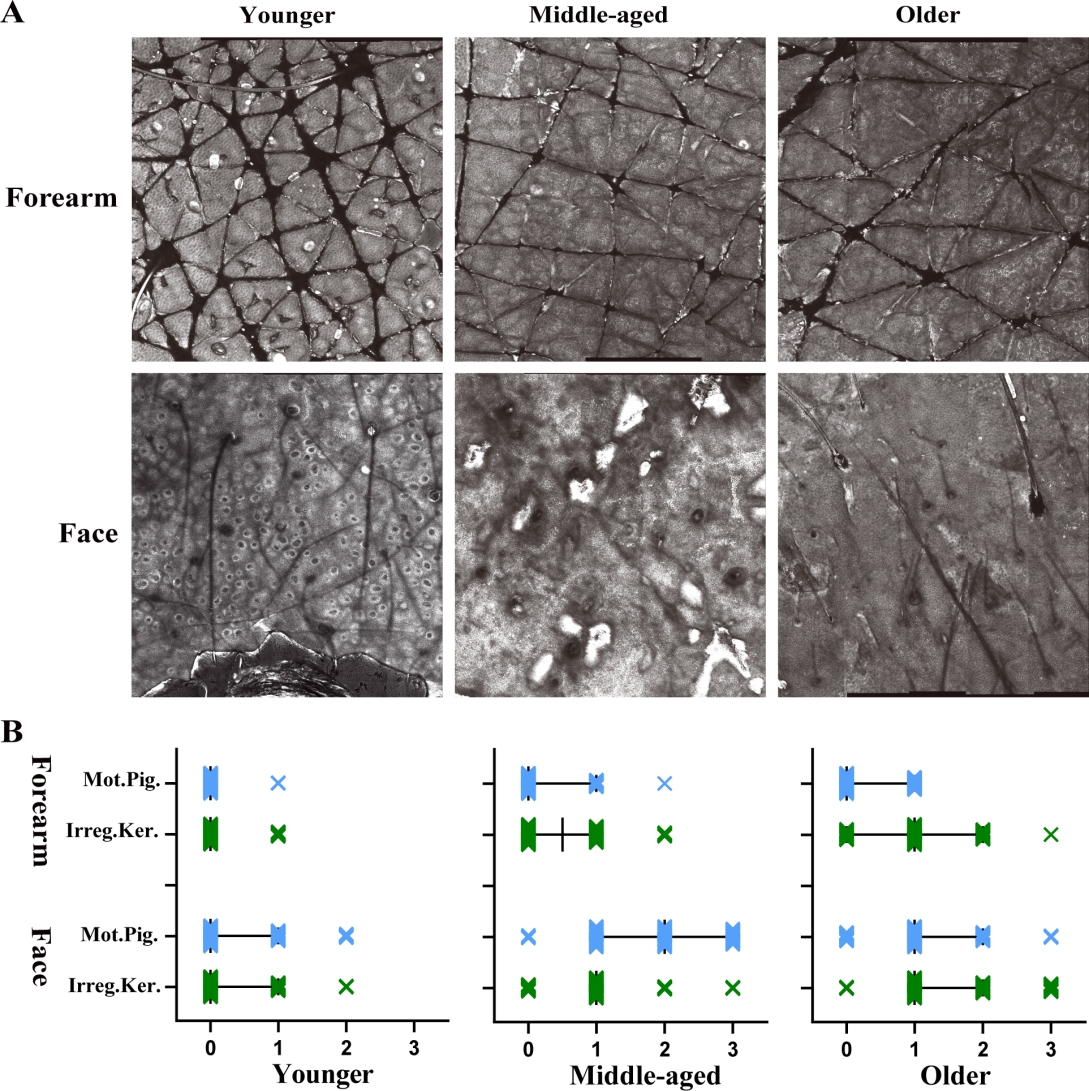
Irregular honeycomb pattern (Irreg. ker.) and Mottled pigmentation (Mot. pig.). **(A)** Representative RCM images showing minimal irregularity in younger subjects, prominent mottled pigmentation in middle-aged facial skin, and extensive irregular honeycomb patterns in older subjects. **(B)** Distribution charts showing the prevalence and extent of irregular honeycomb patterns and mottled pigmentation across age groups and anatomic sites (n = 50 per group). X-axis = semiquantitative score (0–3) indicating feature extent; higher scores indicate a more extensive involvement. Each “×” represents an individual data point; central markers show the median, and error bars indicate the interquartile range (IQR).

The prevalence of irregular honeycomb patterns differed across age groups at both anatomic sites (face: young 30%, middle 78%, older 94%; forearm: young 20%, middle 50%, older 74%). In the elderly group, extensive involvement (>10% area) was more common in facial skin (46%) compared with forearm skin (26%) (P = 0.0006). Representative examples of irregular honeycomb patterns and mottled pigmentation are shown in **Figure 2**.

### Dermal-epidermal junction morphology

**Figure 3:**
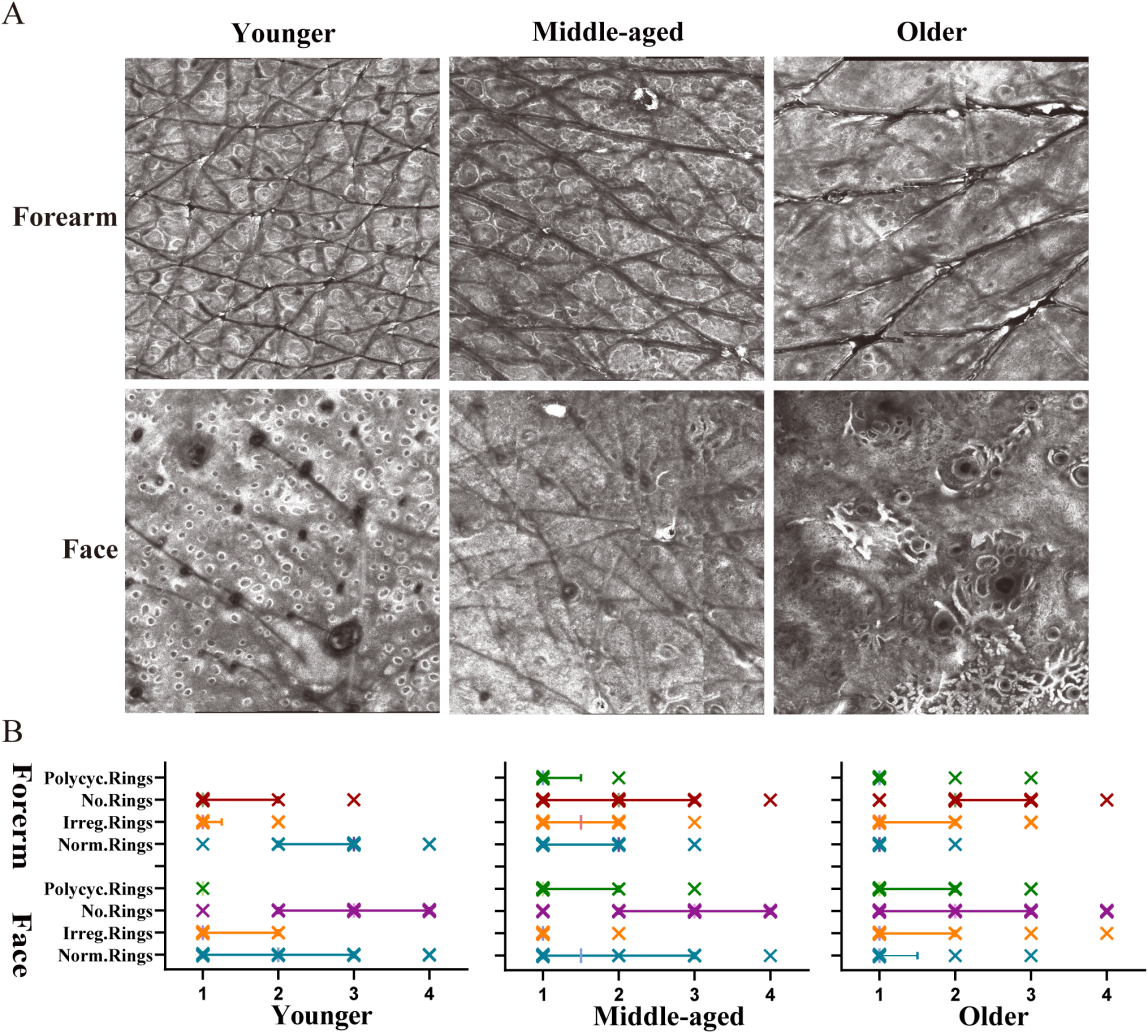
Dermal papillary patterns at the dermoepidermal junction (DEJ). **(A)** Representative images showing regular papillary rings in younger subjects, irregular rings in middle age, and absent rings with polycyclic patterns (arrows) in older facial skin. **(B)** Distribution of papillary ring patterns across age groups and anatomic sites(n = 50 per group). X-axis = coverage-based semiquantitative score (1–3) indicating feature extent; higher scores indicate more extensive coverage. Each “×” represents an individual data point; central markers show the median, and error bars indicate the interquartile range (IQR). Pattern categories: Regular rings (Norm. rings), irregular rings (Irreg. rings), polycyclic rings (Polycyc. rings), and without visible papillary rings (No rings).

Regular papillary rings were more prevalent in younger groups (forearm: young 96%, middle-aged 54%, older 24%; face: young 40%, middle-aged 32%, older 18%). Polycyclic rings were observed on facial skin across age strata (young, 2%; middle-aged, 32%; older, 50%). Papillary ring scores differed across age groups at both sites. On the forearm, median scores were 1.00 (1.00–2.00) in the young, 5.00 (3.00–7.00) in middle-aged, and 6.50 (5.00–8.00) in older group. Scores were significantly higher in older and middle-aged compared with younger (both P < 0.0001, middle-aged vs. younger, r = 0.49; older vs. younger, r = 0.73), higher in older than middle-aged participants (P = 0.010, r = 0.24). On the face, median scores were 6.50 (4.00–8.00) in the young group, 7.00 (6.00–8.00) in the middle-aged group, and 8.00 (6.00–9.00) in the older group. No significant difference was found between young and middle−aged groups (P = 0.372). The older group had significantly higher scores than the young group (P = 0.0001, r = 0.33) and middle-aged participants (P = 0.034, r = 0.21). Examples of papillary ring patterns are shown in **Figure 3**.

### Dermal matrix degradation

**Figure 4:**
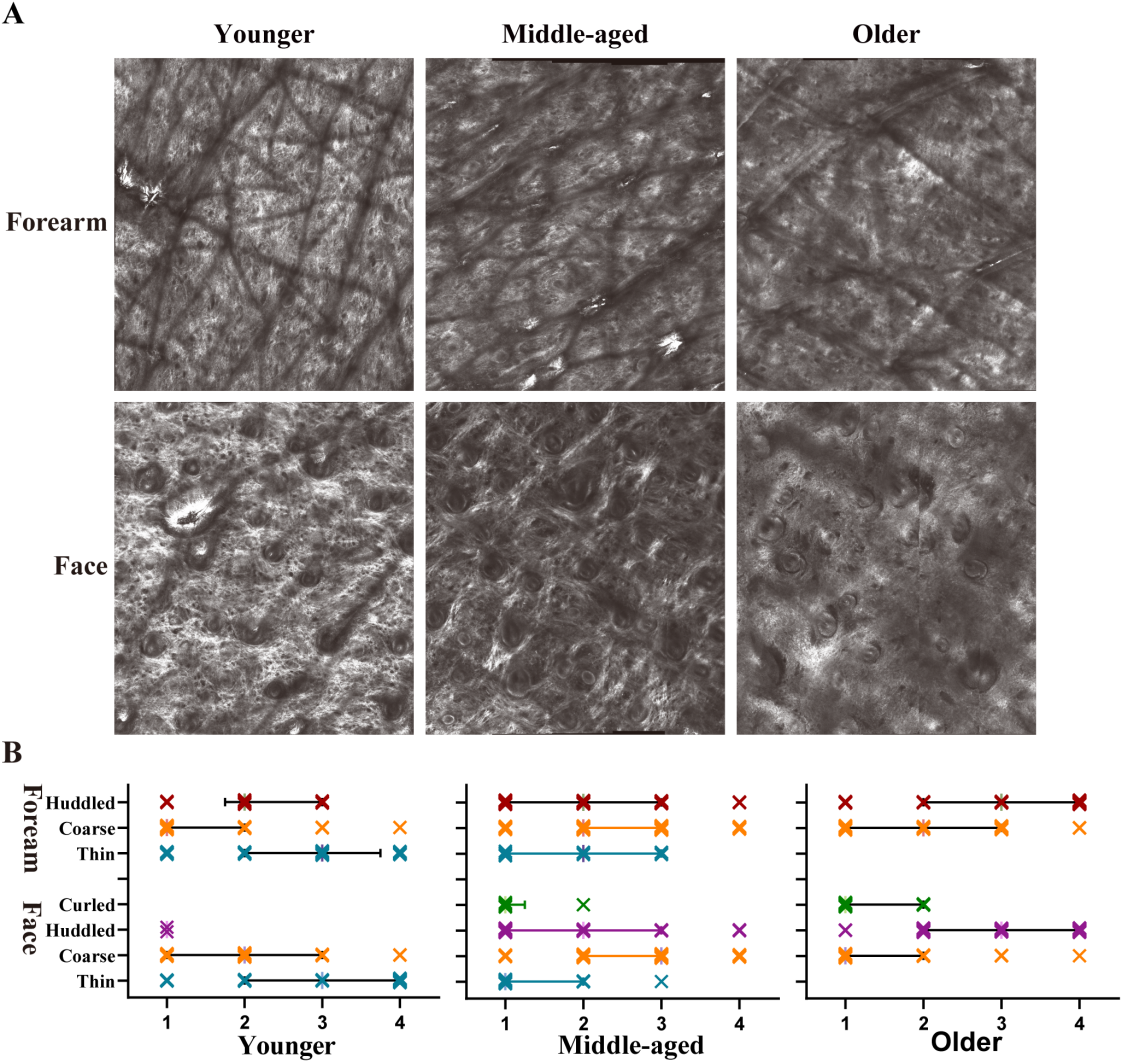
Dermal collagen fiber patterns. **(A)** Representative RCM images demonstrating thin reticulated collagen in younger subjects, coarse collagen in middle age, and huddled/curling collagen in older subjects. Note the presence of curled collagen (arrows) only in older facial skin. **(B)** Distribution charts of collagen types across age groups and anatomic sites (n = 50 per group). X-axis = semiquantitative score (1–4) indicating feature extent; higher scores indicate more extensive coverage. Each “×” represents an individual data point; central markers show the median, and error bars indicate the interquartile range (IQR).

Collagen phenotypes differed across age groups and sites. Thin reticulated patterns predominated in young facial skin (96%). Coarse collagen was more prevalent in middle-aged skin at both sites (face 88%, forearm 94%). Curled collagen, characteristic of solar elastosis, was observed in older facial skin (36%) but was absent in all forearm samples. Dermal collagen fiber patterns are shown in **Figure 4**.

A significant site-by-age interaction was observed for collagen scores. In the young group, forearm scores were higher than facial scores (forearm: 2.000 [1.000–4.000] vs. face: 1.00 [0.000–2.000]; P = 0.0003). In the middle-aged group, facial and forearm scores were comparable (P = 0.21). In the elderly group, the median facial collagen score was significantly higher than the median forearm collagen score (face: 8.000 [7.000–8.250] vs. forearm: 7.000 [6.000–8.000]; P = 0.002). Both for facial and forearm sites, with significant between-group differences observed across young, middle-aged, and elderly groups (all P < 0.0001).

### The effect of work environment

Occupational UV exposure significantly affected facial skin but not forearm skin, with more pronounced differences observed in ≥45 age groups. Among middle-aged participants, indoor workers showed lower epidermal thickness (46.3 ± 7.97 μm) compared with outdoor workers (53.1 ± 7.37 μm; P = 0.004), as well as less extensive mottled pigmentation (indoor 1.500 [1.000–2.000] vs. outdoor 2.000 [1.250–3.000]; P = 0.020) and lower collagen scores (indoor 4.000 [3.000–5.250] vs. outdoor 6.000 [4.250–7.000]; P = 0.011). Among older participants, outdoor workers showed higher collagen scores (outdoor 8.000 [8.000–9.000] vs. indoor 7.000 [5.750–8.000]; P = 0.003), higher papillary ring scores (outdoor 8.000 [7.250–9.000] vs. indoor 7.000 [5.00–9.000]; P = 0.020), and more extensive irregular honeycomb (outdoor 2.000 [1.000–2.750] vs. indoor 1.000 [1.000–2.000], P = 0.043) compared with indoor workers.

### Correlation analysis

**Figure 5:**
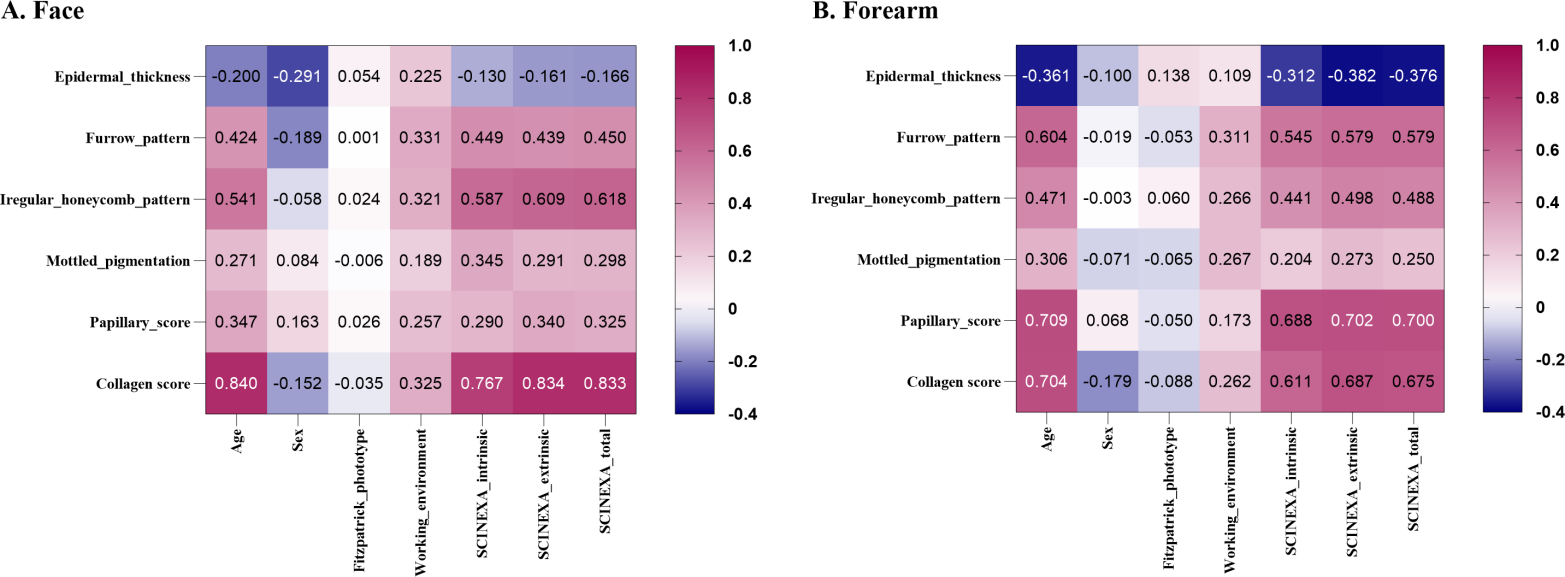
Correlation heatmaps between skin aging features and clinical parameters. **(A)** Facial skin. **(B)** Volar forearm skin. Color intensity and direction indicate the strength and polarity of correlations (red, positive; blue, negative).

Facial collagen score demonstrated the strongest correlation with chronological age (r = 0.840, P<0.001), outperforming ET (r = -0.200, P = 0.018). Irregular honeycomb and papillary scores correlated with both intrinsic (r = 0.587, r = 0.290) and extrinsic (r = 0.609, r = 0.340) SCINEXA subscales. Female sex correlated with thinner ET (r = -0.291, P = 0.002) but not with collagen parameters. Correlations between skin aging features and clinical parameters are shown in **Figure 5**.

### Analysis of histopathological

**Figure 6:**
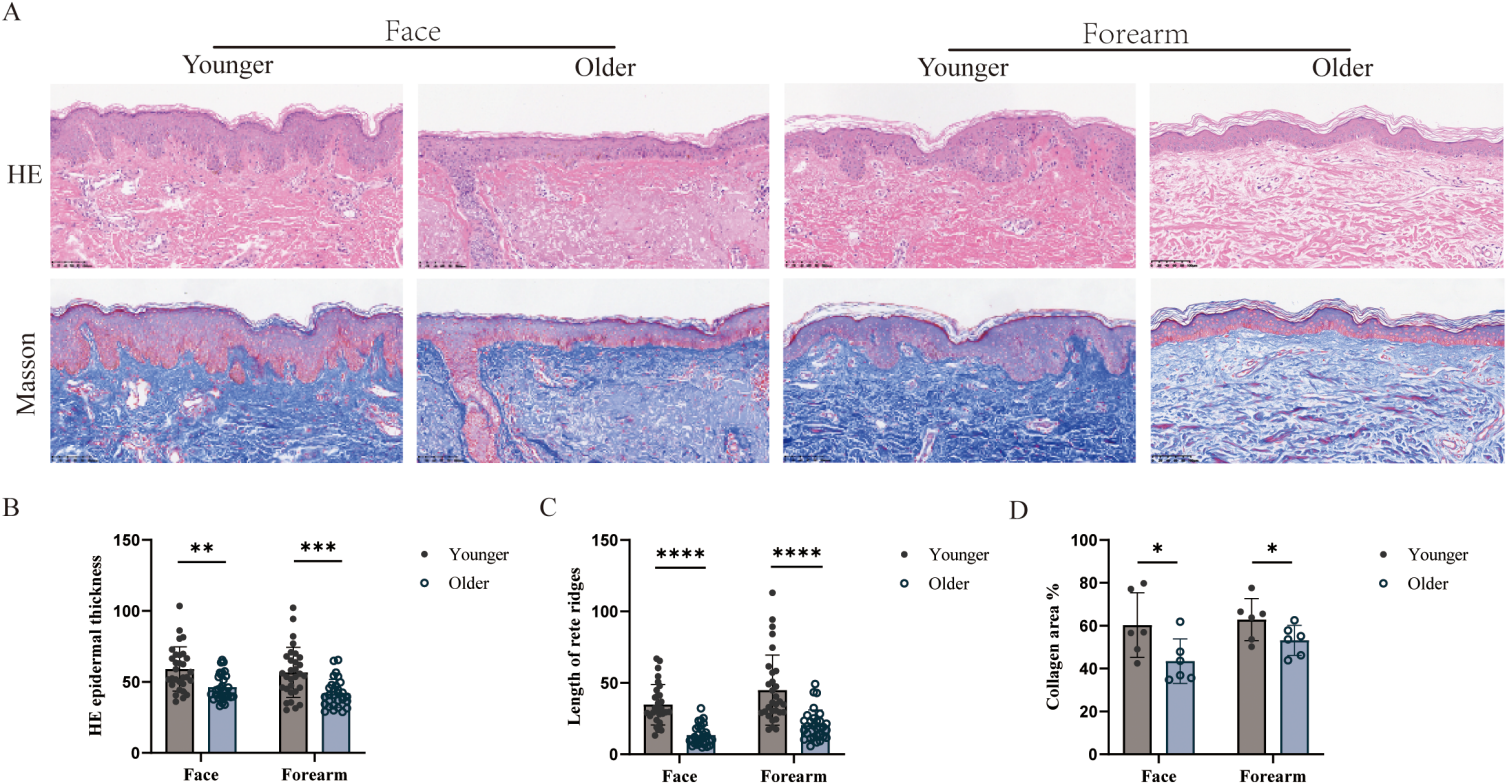
Histopathological characteristics and quantitative analysis of facial and forearm skin in young and elderly subjects. **(A)** Representative H&E and Masson’s trichrome staining images of facial and forearm skin from young and elderly groups (scale bar: 200 μm). **(B)** Epidermal thickness quantification shows significant thinning in the elderly versus young groups, with a more pronounced reduction in the forearm. **(C)** Rete ridge length quantification demonstrates significant shortening in elderly groups with a greater reduction in facial skin. **(D)** Collagen-positive area percentage quantification by Masson’s trichrome shows a significant decrease in elderly groups. **(B–D)** Quantitative data are presented as individual data points with error bars representing mean ± standard deviation (SD). Statistical significance: *P < 0.05, **P < 0.01, ***P < 0.001, ****P < 0.0001.

H&E and collagen staining revealed distinct patterns across groups and anatomical sites (**Figure 6A**). Young subjects exhibited thicker epidermis with longer epidermal rete ridges at both facial (sun-exposed) and forearm (sun-protected) sites, whereas elderly subjects showed epidermal atrophy with shortened or effaced epidermal rete ridges (P < 0.001) (**Figures 6B, C**). Notably, facial skin demonstrated prominent basophilic degeneration in the superficial dermis with disorganized and fragmented collagen fibers, while forearm skin exhibited only overall collagen sparsity without basophilic changes. Quantitative analysis of Masson’s staining showed a lower collagen-positive area in older compared with young participants at both facial (P = 0.047) and forearm sites (P = 0.049) (**Figure 6D**).

## Discussion

Genetic diversity, geographic location, and lifestyle variations contribute to population-specific differences in skin aging morphology (Ali et al., 2024; Jablonski, 2013), necessitating individualized prevention and treatment strategies. As the Chinese aesthetic medicine market expands swiftly, non-invasive pre- and post-treatment evaluations are crucial for directing anti-aging strategies. However, limited RCM data exist regarding healthy Chinese skin. This study employed RCM—validated by concurrent histological examination (H&E and Masson’s trichrome staining)—to characterize site-specific aging patterns in facial (photoexposed) and volar forearm (sun-protected) skin across three age groups in healthy adults from Southwest China. In this cross-sectional cohort, age-group differences suggested distinct intrinsic versus photoaging patterns, site-dependent collagen variation, and potential occupational UV influences. These observations provide regional reference data for understanding skin aging phenotypes in Chinese, with broader generalizability requiring multi-center validation.

Consistent with previous reports (Chen et al., 2023), facial epidermis was thicker than volar forearm epidermis across all age groups in our cohort, also aligning with systematic review data showing greater cheek thickness (86.3 μm) versus volar forearm (80.2 μm) and dorsal forearm (70.3 μm) in Asian populations (Lintzeri et al., 2022). We observed that volar forearm epidermal thickness was comparable between young and middle-aged groups, with lower values in the older group. This pattern in sun-protected skin is compatible with intrinsic aging hypotheses, where structural differences are more pronounced between younger and older age strata. Alternatively, this distribution may reflect cohort-specific differences in nutritional status, occupational patterns, or skincare practices across birth cohorts. Facial skin showed a different pattern across age strata, with higher thickness in the middle-aged group compared with both young and older groups, consistent with Longo et al (Longo et al., 2013), but discrepant from some reports possibly due to ethnic or environmental variation (Kutlu Haytoglu et al., 2014; Lupu et al., 2022). Histopathological examination showed findings similar to these RCM observations, with a smaller magnitude of epidermal thickness difference between young and older groups on the face than on the forearm. These site-specific patterns are compatible with the superimposition of photoaging effects on intrinsic aging, though alternative explanations including cohort-specific UV exposure differences cannot be excluded.

Furrow patterns showed similar age-stratified patterns at both sites: small rhomboidal patterns predominant in youth, while large/disarranged furrows and linear patterns were more prevalent in older groups. Due to anatomic differences, absent furrows were observed only on facial skin (occurring across all age groups). Stamatas et al (Stamatas et al., 2010). reported that infant skin exhibits more numerous wrinkles, forming smaller skin islands. Whether the absent furrow pattern observed on facial skin across all age strata is associated with chronic UV exposure–induced effacement remains speculative. Longitudinal studies including pediatric and adolescent cohorts are required to distinguish developmental, environmental, and technical contributions. Mottled pigmentation represents a primary perceived aging feature in Chinese subjects aged 20–30 years (Quan et al., 2024). We observed peak pigmentation in middle age (94% prevalence; 60% with >10% coverage), consistent with chronic UV-induced melanocyte hyperactivity (Gilchrest, 1982). However, pigmentation declined in older ages despite continued UV exposure, likely due to age-related melanocyte degeneration and reduced enzymatically active melanocyte numbers after age 61, resulting in decreased melanogenesis (Kang et al., 2021; Kim et al., 2022; Yaar and Gilchrest, 2001). Though cohort-specific differences in sun exposure history, cosmetic product use, or selection effects among older volunteers cannot be excluded. Some studies report continued pigmentary increase with age, highlighting the need for further investigation. Irregular honeycomb patterns, corresponding histologically to altered keratinocyte morphology and loss of polarity, increased progressively with age at both sites. Previous studies have linked extensive irregular honeycomb patterns (>10% area) to a history of non-melanoma skin cancer (Lagarrigue et al., 2012). Whether this association reflects photo-induced keratinocyte damage, cumulative UV exposure, or other factors requires further investigation.

Regular papillary rings were more prevalent in the young group at both sites, while irregular patterns and ring absence were more common in older groups. This cross-sectional pattern is compatible with age-associated flattening of the dermo-epidermal junction, though technical factors (small field of view, anatomic curvature) and cohort-specific UV exposure histories cannot be excluded. Histopathology showed more pronounced rete ridge shortening in facial versus forearm skin, consistent with UV-accelerated junctional effacement (Curiel-Lewandrowski et al., 2021; Russell-Goldman and Murphy, 2020). Notably, absent papillary rings were observed across all ages on facial skin, potentially attributable to anatomic factors and phototype-related differences, previous studies report that Fitzpatrick phototypes I-II less frequently demonstrate regular papillary rings on facial skin (Lintzeri et al., 2022). Polycyclic rings emerged as a specific photoaging marker on sun-exposed facial skin, more prevalent on sun-exposed facial skin in older groups (young, 4% vs. older, 50%), though some studies report peak prevalence in middle age with a subsequent decline (Kligman, 1969).

This superficial damage was paralleled by dermal collagen remodeling. Dermal collagen showed age-group and site-specific patterns. Thin reticulated collagen typified youthful dermis, while coarse and huddled patterns were more common in older groups. Masson’s trichrome staining shows similar results to RCM collagen scores: sparse, fragmented fibers with decreased staining intensity in older age, more pronounced on the face with basophilic degeneration—consistent with RCM’s utility for quantitative collagen assessment. Notably, collagen scores revealed a site-specific pattern: the forearm showed higher values than the face in the young group, while the face showed higher values than the forearm in middle-aged and older groups. This cross-sectional distribution is compatible with differential photoaging exposure across anatomic sites (Robertson and Rees, 2010; Watson et al., 1999), though baseline anatomic differences and cohort-specific UV histories cannot be excluded. This extracellular matrix deterioration underlies the observed DEJ flattening and furrow effacement, completing the photoaging cascade from superficial keratinocyte damage to deep dermal degeneration.

Work environment analysis revealed structural differences between outdoor and indoor workers. Outdoor workers resulted in thicker facial epidermis, higher collagen scores, and more disarranged/linear furrow patterns in middle-aged and older subjects. Middle-aged outdoor workers showed more extensive mottled pigmentation, while older outdoor workers showed more extensive irregular honeycomb patterns and higher papillary ring scores. These cross-sectional patterns are compatible with UV exposure-associated structural alterations, including epidermal thickening, collagen changes, DEJ remodeling, and altered keratinocyte morphology. The observed collagen patterns coincide with photoaging-associated structural alterations described in previous studies (Amano, 2016; Kaltchenko and Chien, 2025), including matrix metalloproteinase activity increase and solar elastosis. Outdoor workers demonstrated accelerated facial collagen loss and more flattened dermal papillae histologically, consistent with previous reports of matrix metalloproteinase activity as a UV-associated effector mechanism. Causal mechanisms cannot be inferred from this cross-sectional comparison. The RCM-histology concordance observed in this cohort supports the feasibility of non-invasive collagen assessment; however, validation as a clinical tool for photoprevention monitoring requires prospective intervention studies. Interestingly, work environment did not significantly affect volar forearm skin at any age or facial skin in younger subjects. This absence of group differences may reflect lower cumulative UV exposure in these strata, though selection effects and measurement variability cannot be excluded. The comparable findings in young facial skin across work environments suggest that detectable structural differences may require more prolonged exposure, emphasizing the importance of early photoprotection initiation. This study represents the first quantitative assessment of papillary ring patterns and confirms their good correlation with SCINEXA.

This single-center cross-sectional study describes age-group differences that reflect between-subject comparisons across birth cohorts rather than intra-individual aging trajectories. Childhood UV exposure, nutritional status, sun protection behaviors, and occupational patterns are inextricably confounded with chronological age, precluding causal inference. The Fitzpatrick phototype distribution was predominantly type III, with limited type II and IV, restricting generalizability to lighter or darker skin phenotypes. The binary work-environment classification (outdoor vs. indoor) is a crude proxy that does not capture cumulative UV dose, seasonal variation, or individual protective behaviors. Histological validation was limited by small sample size and lack of matched forearm-face specimens from the same individuals. Future studies require multi-center recruitment, quantitative UV dosimetry, comprehensive lifestyle covariates (diet, smoking, sleep, BMI, etc.), longitudinal intra-individual designs with repeated assessments, and prospective intervention trials to validate these cross-sectional observations and establish true aging trajectories.

In conclusion, this study provides the first comprehensive RCM characterization of healthy Chinese cohort skin across ages and anatomic sites, with histological and collagen staining validation in a subset. Our results describe age-group differences in irregular honeycomb structures, collagen patterns, and forearm epidermal thickness that are observationally consistent with patterns reported in other populations. Facial epidermal thickness, polycyclic papillary rings, and mottled pigmentation showed population differences that require cross-population and multicenter validation. RCM-detected structural changes exhibited concordance with histopathological findings in the examined subset. This observational concordance supports the feasibility of non-invasive collagen assessment and provides baseline reference ranges for future studies. Though clinical utility for photoprevention monitoring requires prospective intervention validation. Outdoor workers showed greater structural alterations in middle-aged and older groups compared with indoor workers, consistent with cumulative UV exposure effects, though the specific exposure threshold for detectable changes remains undefined. Longitudinal studies with quantitative UV dosimetry are needed to establish dose-response relationships and validate the optimal timing of preventive intervention.

## Data Availability

The raw data supporting this article are available upon reasonable request and with the approval of the corresponding author.
